# Supplementary material for: Overcoming barriers to data sharing with medical image generation: a comprehensive evaluation
Source: NPJ Digit Med. 2021 Sep 24;4:141. doi: 10.1038/s41746-021-00507-3 (PMC8463544; doi:10.1038/s41746-021-00507-3)
Supplement: Supplementary file 1 — Reporting summary. [file 41746_2021_507_MOESM1_ESM.pdf]

nature portfolio

Corresponding author(s): August DuMont Schütte
Last updated by author(s): Jun 16, 2021

Reporting Summary

Nature Portfolio wishes to improve the reproducibility of the work that we publish. This form provides structure for consistency and transparency in reporting. For further information on Nature Portfolio policies, see our Editorial Policies and the Editorial Policy Checklist.

Statistics

For all statistical analyses, confirm that the following items are present in the figure legend, table legend, main text, or Methods section.

n/a	Confirmed	
☐	☒	The exact sample size (*n*) for each experimental group/condition, given as a discrete number and unit of measurement
☐	☒	A statement on whether measurements were taken from distinct samples or whether the same sample was measured repeatedly
☐	☒	The statistical test(s) used AND whether they are one- or two-sided<br>*Only common tests should be described solely by name; describe more complex techniques in the Methods section.*
☒	☐	A description of all covariates tested
☐	☒	A description of any assumptions or corrections, such as tests of normality and adjustment for multiple comparisons
☐	☒	A full description of the statistical parameters including central tendency (e.g. means) or other basic estimates (e.g. regression coefficient) AND variation (e.g. standard deviation) or associated estimates of uncertainty (e.g. confidence intervals)
☐	☒	For null hypothesis testing, the test statistic (e.g. *F*, *t*, *r*) with confidence intervals, effect sizes, degrees of freedom and *P* value noted<br>*Give P values as exact values whenever suitable.*
☒	☐	For Bayesian analysis, information on the choice of priors and Markov chain Monte Carlo settings
☒	☐	For hierarchical and complex designs, identification of the appropriate level for tests and full reporting of outcomes
☒	☐	Estimates of effect sizes (e.g. Cohen's *d*, Pearson's *r*), indicating how they were calculated

Our web collection on statistics for biologists contains articles on many of the points above.

Software and code

Policy information about availability of computer code

Data collection

Provide a description of all commercial, open source and custom code used to collect the data in this study, specifying the version used OR state that no software was used.

Data analysis

Python3 Packages:
numpy==1.13.3, tensorflow-gpu==1.14.0, tensorflow==1.14.0, keras==2.2.4, scikit-image==0.16.2, scikit-learn==0.22.1, six==1.11.0, h5py==2.10.0, pillow==3.1.1, scipy==1.0.0, lmdb==0.93, opencv-python==3.4.0.12, cryptography==2.1.4, pandas==0.23.4

GitHub Repositories:
(1) Tero Karras, NVIDIA CORPORATION, 2018: https://github.com/tkarras/progressive_growing_of_gans
(2) Bruce Chou, 2018: https://github.com/brucechou1983/CheXNet-Keras
(3) Patrick Schwab, 2019: https://github.com/d909b/cxplain

For manuscripts utilizing custom algorithms or software that are central to the research but not yet described in published literature, software must be made available to editors and reviewers. We strongly encourage code deposition in a community repository (e.g. GitHub). See the Nature Portfolio guidelines for submitting code & software for further information.

Data

Policy information about availability of data

All manuscripts must include a data availability statement. This statement should provide the following information, where applicable:

- Accession codes, unique identifiers, or web links for publicly available datasets
- A description of any restrictions on data availability
- For clinical datasets or third party data, please ensure that the statement adheres to our policy

Both datasets are open-source and free to download for any registered user:
(1) CheXpert: https://stanfordmlgroup.github.io/competitions/chexpert/
(2) RSNA Intracranial Hemorrhage Detection: https://www.kaggle.com/c/rsna-intracranial-hemorrhage-detection

Field-specific reporting

Please select the one below that is the best fit for your research. If you are not sure, read the appropriate sections before making your selection.

☒ Life sciences ☐ Behavioural & social sciences ☐ Ecological, evolutionary & environmental sciences

For a reference copy of the document with all sections, see nature.com/documents/nr-reporting-summary-flat.pdf

Life sciences study design

All studies must disclose on these points even when the disclosure is negative.

Sample size	Chest x-ray dataset: 147,173 x-rays from 55,192 patients<br>Brain CT dataset: 215,866 CT scans from 18,917 patients
Data exclusions	All lateral chest x-rays were excluded. All chest x-rays with label combinations that appear less than 256 times in the dataset were excluded. All brain CT scans with label combinations that appear less than 100 times in the dataset were excluded. Brain CT scans with no radiology finding were under-sampled to achieve a balanced dataset where 50% of brain CT scans show some type of hemorrhage.
Replication	The public code repository contains all files to (1) pre-process and transform the raw datasets into the exact experimental settings, (2) train, validate and test all generative and predictive models and (3) perform all post-processing analysis
Randomization	After data exclusion both datasets were randomly split, on a patient-level and within strata of radiology findings into the training set (80%), validation set (10%) and test set (10%)<br>For each reader study 100 synthetic test images were randomly sampled, after which 100 real test images with the same label combinations were randomly sampled.
Blinding	For blinding during the data collection please refer to the open-source datasets.<br>For each reader study, participants labeled images as real or synthetic without knowledge of any clinical information from the patients and without knowing the radiology finding for the particular image.

Reporting for specific materials, systems and methods

We require information from authors about some types of materials, experimental systems and methods used in many studies. Here, indicate whether each material, system or method listed is relevant to your study. If you are not sure if a list item applies to your research, read the appropriate section before selecting a response.

Materials & experimental systems

n/a	Involved in the study	
☒	☐	Antibodies
☒	☐	Eukaryotic cell lines
☒	☐	Palaeontology and archaeology
☒	☐	Animals and other organisms
☒	☐	Human research participants
☒	☐	Clinical data
☒	☐	Dual use research of concern

Methods

n/a	Involved in the study	
☒	☐	ChIP-seq
☒	☐	Flow cytometry
☒	☐	MRI-based neuroimaging
