# Supplementary material for: Overcoming barriers to data sharing with medical image generation: a comprehensive evaluation
Source: NPJ Digit Med. 2021 Sep 24;4:141. doi: 10.1038/s41746-021-00507-3 (PMC8463544; doi:10.1038/s41746-021-00507-3)
Supplement: Supplementary file 2 — Supplementary information. [file 41746_2021_507_MOESM2_ESM.pdf]

Supplementary Information

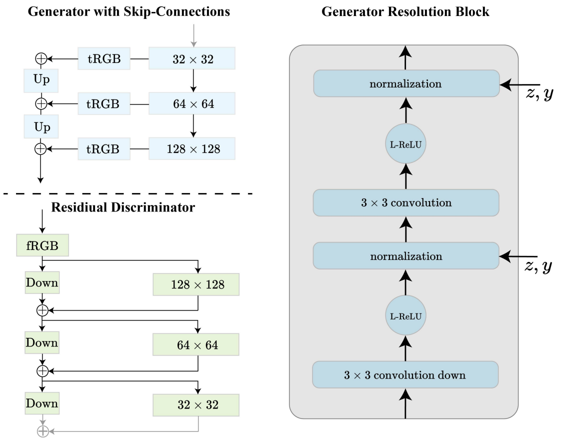

Supplementary Figure 1: **Network architecture and generator block. Left:** In the generator, output skip connections in the image feature space are included after each resolution block, while the discriminator blocks have residual connections. *Up* and *Down* refer to nearest neighbour upsampling and downsampling by average pooling while *tRGB* and *fRGB* refer to the 1×1 convolution mappings to and from the image space. **Right:** The first convolution in each generator block doubles the spatial resolution via nearest neighbour upsampling and reduces the number of feature channels (if needed). Each pixel-wise feature vector normalisation layer is conditioned on the label information $\mathbf{y}$ and random normal noise vector $\mathbf{z}$. The Leaky-ReLU non-linearity is used as an activation function.

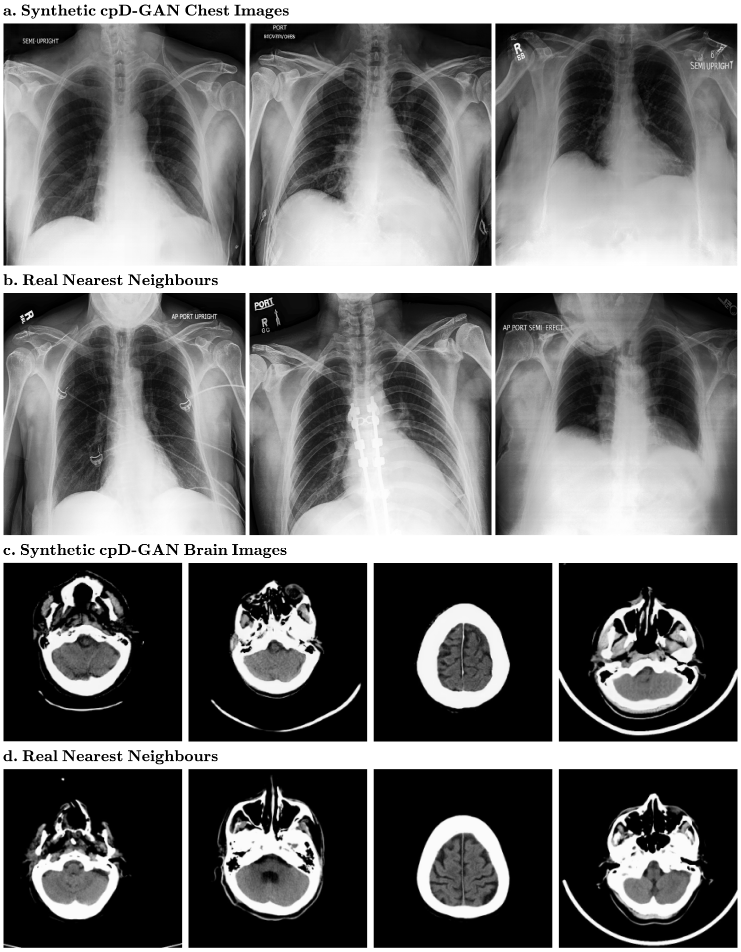

Supplementary Figure 2: **More randomly sampled synthetic images generated by the cpD-GAN and real nearest neighbour images from the training. a)** Synthetic chest radiographs at 512×512 pixels. **b)** Nearest matching real images found in the chest radiograph training set. **c)** Synthetic brain computed tomography (CT) scans at 256×256 pixels. **d)** Nearest matching real images found in the brain CT training set.

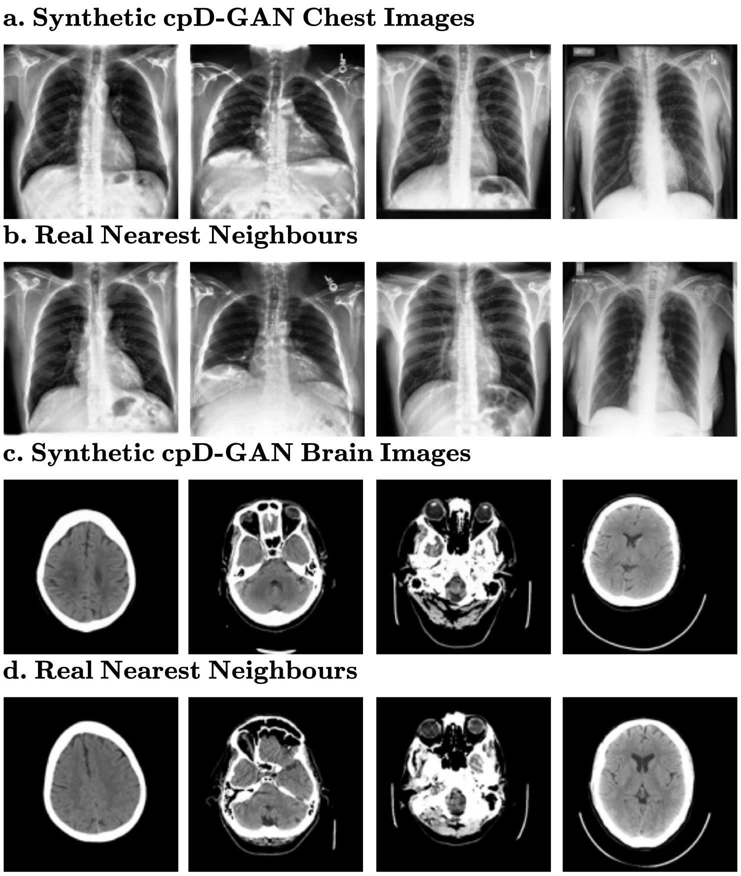

Supplementary Figure 3: **More randomly sampled synthetic images from the cpD-GAN and nearest neighbours from all real training images at a resolution of** 128×128 **pixels.** **a)** Synthetic chest radiographs. **b)** Nearest matching real images found in the chest radiograph training set. **c)** Synthetic brain computed tomography (CT) scans. **d)** Nearest matching real images found in the brain CT training set.

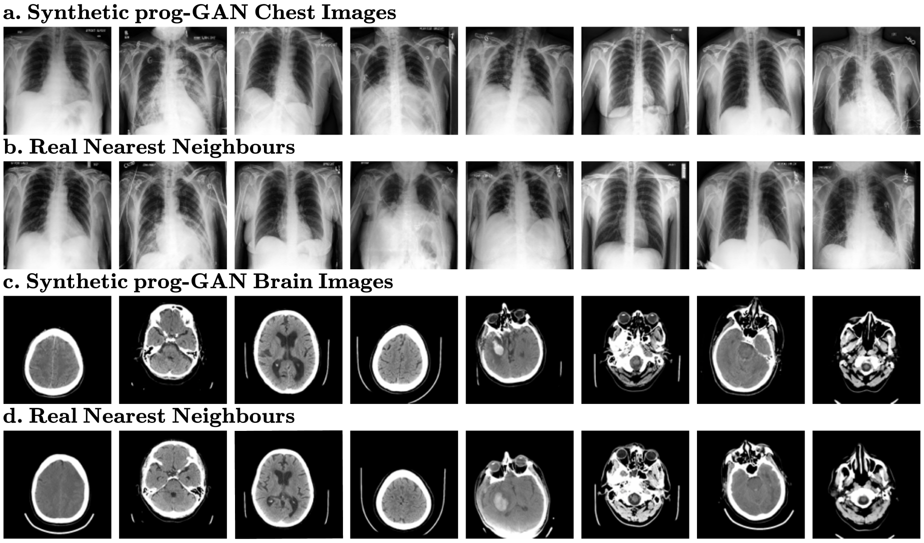

Supplementary Figure 4: **Randomly sampled synthetic images from the prog-GAN and nearest neighbours from all real training images at resolution of** 128×128 **pixels. a)** Synthetic chest radiographs. **b)** Nearest matching real images found in the chest radiograph training set. **c)** Synthetic brain computed tomography (CT) scans. **d)** Nearest matching real images found in the brain CT training set.

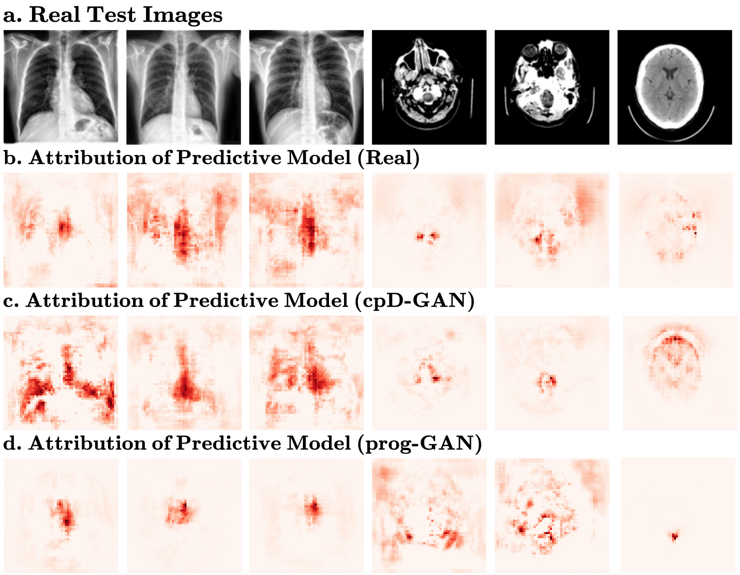

Supplementary Figure 5: **More feature importance maps of predictive models.** Deeper red colour indicates regions that have a larger causal contribution to the label prediction. **a)** Real test images at 128×128 resolution. All displayed images are without any clinical finding. **b)** Feature importance of predictive model trained on real data. **c)** Feature importance of predictive model trained on synthetic data generated by the cpD-GAN. **d)** Feature importance of predictive model trained on synthetic data generated by the prog-GAN.

		**Radiologist**	
		Real	Synthetic
**Actual**	Real	$TR = 25.6$ (± 7.1)	$FS = 24.4$ (± 7.1)
	Synthetic	$FR = 31.0$ (± 8.2)	$TS = 19.0$ (± 8.2)

Accuracies of Radiologist Labels

0.45	0.52	0.45	0.52	0.50	0.25	0.49	0.39	0.40	0.55	0.39

Supplementary Table 1: **Chest radiographs reader study at** 128×128 **pixels resolution. Top:** Means and standard deviation from 11 trained radiologists for real and synthetic images: TR = True Reals, FR = False Reals, TS = True Synthetics, FS = False Synthetics. **Bottom:** Computed accuracies from radiologist labels.

		**Radiologist**	
		Real	Synthetic
**Actual**	Real	$TR = 25.2\ (\pm 3.5)$	$FS = 24.8\ (\pm 3.5)$
	Synthetic	$FR = 30.3\ (\pm 5.8)$	$TS = 19.7\ (\pm 5.8)$

Accuracies of Radiologist Labels

0.51	0.45	0.44	0.44	0.46	0.50	0.48	0.36	0.40

Supplementary Table 2: **Brain CT scans reader study at** 128×128 **pixels resolution. Top:** Means and standard deviation from 9 trained radiologists for real and synthetic images: TR = True Reals, FR = False Reals, TS = True Synthetics, FS = False Synthetics. **Bottom:** Computed accuracies from radiologist labels.

		**Radiologist**	
		Real	Synthetic
**Actual**	Real	$TR = 37.4\ (\pm 5.7)$	$FS = 12.6\ (\pm 5.7)$
	Synthetic	$FR = 16.4\ (\pm 12.5)$	$TS = 33.6\ (\pm 12.5)$

**Accuracies of Radiologist Labels**	0.76	0.46	0.75	0.54	0.93

Supplementary Table 3: **Chest radiographs reader study at** 512×512 **resolution. Top:** Means and standard deviation from 5 trained radiologists for real and synthetic images: TR = True Reals, FR = False Reals, TS = True Synthetics, FS = False Synthetics. **Bottom:** Computed accuracies from radiologist labels.

		**Radiologist**	
		Real	Synthetic
**Actual**	Real	$TR = 31.6\ (\pm 7.6)$	$FS = 18.4\ (\pm 7.6)$
	Synthetic	$FR = 28.4\ (\pm 8.3)$	$TS = 21.6\ (\pm 8.3)$

**Accuracies of Radiologist Labels**	0.54	0.46	0.75	0.54	0.37

Supplementary Table 4: **Brain CT scans reader study at** 256×256 **pixels resolution. Top:** Means and standard deviation from 5 trained radiologists for real and synthetic images: TR = True Reals, FR = False Reals, TS = True Synthetics, FS = False Synthetics. **Bottom:** Computed accuracies from radiologist labels.
